# Neuromyelitis Optica and Immune Thrombocytopenic Purpura Treated With Rituximab in a Patient With Combined Neurologic and Hematologic Disorder: A Case Report

**DOI:** 10.7759/cureus.57818

**Published:** 2024-04-08

**Authors:** Mary Remilenne E Rebanal-Ricafrente, Ma. Luisa Gwenn P Tiongson

**Affiliations:** 1 Center for Neurological Sciences, Quirino Memorial Medical Center, Quezon City, PHL; 2 Clinical Neurosciences, Quirino Memorial Medical Center, Quezon City, PHL

**Keywords:** rituximab, pulse steroid therapy, neuromyelitis optic spectrum disorder, immune thrombocytopenia purpura, nmo, immune thrombocytopenic purpura, neuromyelitis optica spectrum disorder (nmosd)

## Abstract

Neuromyelitis optica spectrum disorder (NMOSD) is a rare autoimmune inflammatory demyelinating disease of the central nervous system affecting the optic nerves and spinal cord. Immune thrombocytopenia (ITP), on the other hand, is an autoimmune disorder characterized by a platelet count of <100 in the absence of any known condition that could be associated with thrombocytopenia.

This case report focuses on a 56-year-old female presenting with the unique coexistence of NMOSD and ITP. A 56-year-old woman of Russian descent had a sudden onset of right eye blindness at the age of 24 and was diagnosed with multiple sclerosis. She developed petechial rashes on both lower extremities two weeks before consultation with no associated findings. Cranial MRI revealed multiple nodular and patchy areas of hyperintense signals on T2-weighted/fluid-attenuated inversion recovery without restricted diffusion. A thoracolumbar MRI revealed long segment foci of intramedullary cord non-enhancing abnormal hyperintense signal from T2 to T11. Cerebrospinal fluid aquaporin 4 IgG was negative. A complete blood count revealed platelets of 4 × 10^9^/L, leading to the management of ITP. She was started on methylprednisolone 1 g/day for five days. Her platelet count improved eventually and rashes resolved. Rituximab treatment was initiated at a dose of 1 g on day 1 and day 15. On the 18th day of admission, the Expanded Disability Status Scale and functional score improved to 6.0 from 7.0 upon admission.

## Introduction

Neuromyelitis optica (NMO) is a condition that predominantly targets the optic nerves, brainstem, and spinal cord, often leading to stepwise deterioration in affected individuals [1]. The progressive accumulation of visual, motor, and sensory deficits and bladder dysfunction due to recurrent attacks constitute the natural history of neuromyelitis optica spectrum disorder (NMOSD).

Immune thrombocytopenic purpura (ITP) is a hematologic disorder characterized by low platelet count. According to Dong et al., B-cell-depleting therapies such as rituximab have been widely used in ITP as second-line treatment due to the significant role of B cells in the pathogenesis of ITP [2].

NMOSD linked to aquaporin-4 immunoglobulin G antibodies (AQP4-IgG) necessitates serologic testing for accurate evaluations [3]. It is important to distinguish between seropositive and seronegative AQP4-IgG; however, it is noteworthy that the serological distinction between seronegative and seropositive NMOSD does not significantly impact relapse rates, disease severity, or long-term outcomes [3]. The intricate interplay between the immune system and hematopoietic processes in ITP raises intriguing questions regarding the potential connections between neurologic disorders and hematologic conditions.

Investigating such rare clinical scenarios is essential for unraveling the intricate mechanisms underlying the concurrence of these conditions and identifying potential treatment synergies that are yet to be discovered. By delving into our patient’s comprehensive clinical history, diagnostics, and therapeutic approach, we aim to shed light on the interrelationship between these two distinct yet interconnected disorders, offering insights that could potentially pave the way for innovative therapeutic strategies and improved patient care. In this report, we elucidate the clinical course, diagnostic challenges, and treatment outcomes of this intriguing case, thereby contributing to the evolving understanding of the complex nexus between neurologic and hematologic disorders.

## Case presentation

We present the case of a 56-year-old woman of Russian descent who had experienced several autoimmune diseases. Her medical history included the onset of right eye blindness at the age of 24, following childbirth, and a subsequent diagnosis of multiple sclerosis (MS). Six months later, she developed right-sided weakness, which was exacerbated by stress. Treatment with intravenous methylprednisolone (1 g for five days) resulted in significant improvement. However, she continued to experience recurrent right eye blindness and bilateral lower extremity weakness over the next eight years, leading to her eventual reliance on a wheelchair.

Notably, the patient’s clinical course took an unexpected turn when she developed petechial rashes on both lower extremities two weeks before her consultation. These rashes appeared without any history of bleeding, vomiting, fever, cough, colds, or trauma. In 2018, the patient was diagnosed with multiple gastric ulcers, erosive gastritis, and a *Helicobacter pylori* infection. Additionally, she received a diagnosis of rectosigmoid adenocarcinoma stage I and underwent a sigmoidectomy with anastomosis.

Upon admission, the physical examination revealed the persistence of petechial rashes on both lower extremities. The neurologic evaluation showed reduced visual acuity (20/100 OD and 20/70 OS), with motor examination findings of 4/5 strength on neck flexion, shoulder elevation, shoulder abduction, elbow flexion, wrist and finger extension, as well as in the lower extremities. Sensory testing indicated 70% sensory intact on the right and left T7-L2 and 10% intact on L3-S1 bilaterally. The Expanded Disability Status Scale (EDSS) score upon admission was 7.0.

**Figure 1:**
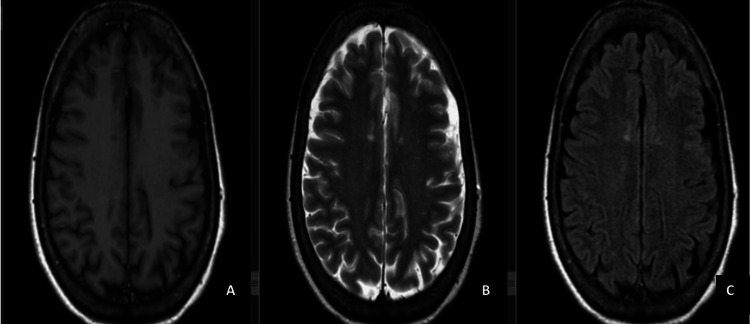
Cranial MRI with T1W (A) and T2W/FLAIR (B, C) hyperintense signals in the left occipital and bilateral temporal convexities. Nodular and patchy areas of T2W/FLAIR hyperintense signals in the centrum semiovale, periventricular white matter, cerebral peduncle, and pons. Small CSF-like signals in the medial temporal lobes. A: T1W. B: T2W. C: FLAIR. T1W: T1-weighted; T2: T2-weighted; FLAIR: fluid-attenuated inversion recovery; CSF: cerebrospinal fluid

Advanced imaging studies were conducted to elucidate the underlying pathologies. Cranial MRI with contrast revealed extra-axial crescentic signals in the left occipital and bilateral temporal convexities. Nodular and patchy areas of hyperintense signals on T2-weighted/fluid-attenuated inversion recovery without restricted diffusion were observed in the bilateral centrum semiovale and periventricular white matter of the frontal and parietal lobes, left cerebral peduncle, and pons (Figure 1).

**Figure 2:**
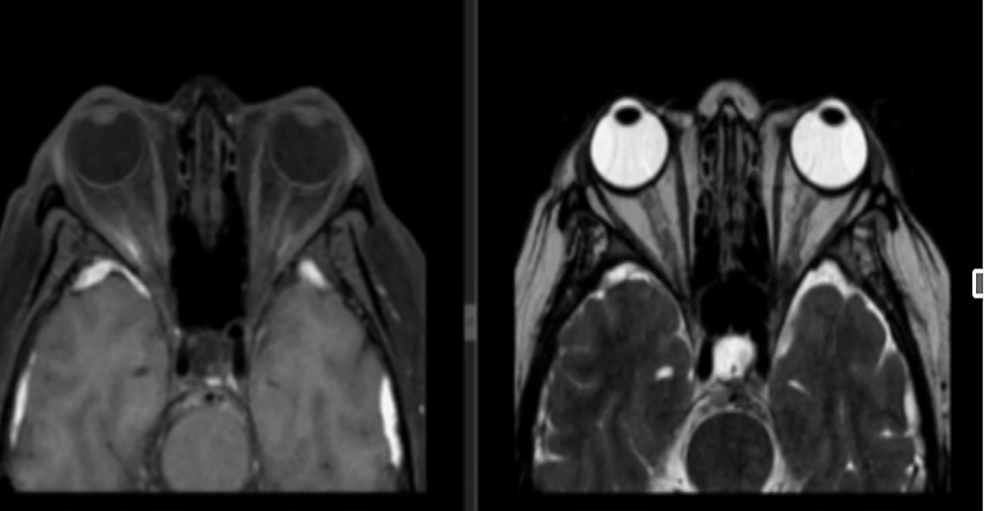
Orbital MRI revealing no distinct mass or enhancing lesions, normal extraconal, intraconal, preseptal, and inferior or infraorbital regions; symmetrical extraocular muscles; and normal-sized optic nerves. Right: T2-weighted. Left: FLAIR: fluid-attenuated inversion recovery.

**Figure 3:**
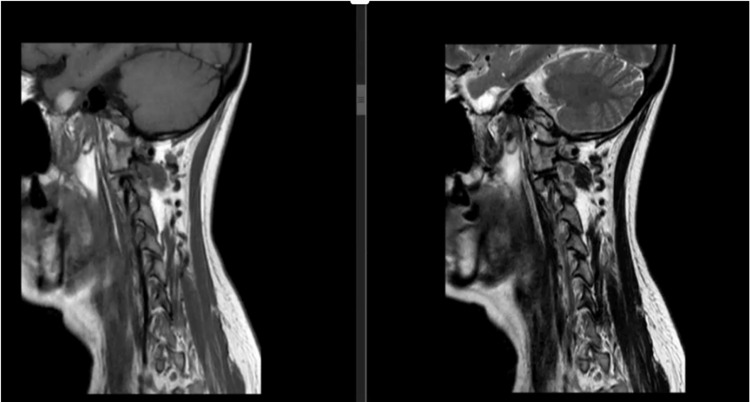
Cervical MRI displaying straightening of the normal cervical lordosis and intrinsically normal cervical cord without abnormal areas of enhancement. Right: T2-weighted. Left: FLAIR: fluid-attenuated inversion recovery.

Small cerebrospinal fluid (CSF)-like signals were noted in the bilateral medial temporal lobes. There was no evidence of acute infarct or abnormal contrast enhancement. Orbital MRI showed no distinct mass or enhancing lesions, (Figure 2), and cervical MRI displayed normal findings with intrinsically normal cervical cord without areas of enhancement (Figure 3).

A thoracolumbar MRI performed in 2021 revealed long segment foci of intramedullary cord non-enhancing abnormal hyperintense signal from T2 to T11, accompanied by mild volume loss. These findings raised the possibility of demyelinating diseases such as NMO rather than MS.

Additionally, the patient’s complete blood count indicated thrombocytopenia with a platelet count of 4 × 10^9^/L, with peripheral blood smear showing few normal-shaped platelets.

CSF testing for CSF AQP4-IgG and anti-myelin oligodendrocyte glycoprotein returned negative results. Given these findings, the patient was managed as a case of double seronegative NMO, with consideration for an underlying diagnosis of ITP.

The patient was promptly referred to a hematologist for co-management and was initiated on pulse therapy with intravenous methylprednisolone (1,000 mg/day) for five days. Remarkably, her platelet count improved from 4 to 50, then to 70, and eventually 100. The petechial rashes on all extremities resolved on the seventh day post-completion of pulse therapy. Subsequently, rituximab treatment was initiated at a dose of 1 g on day 1 and day 15. The patient also exhibited improvement in motor strength, with a transition from 4/5 to 4+/5 in all extremities. On the 18th day of hospitalization, her EDSS improved to 6.0 from the initial score of 7.0 upon admission.

## Discussion

The diagnostic challenges in this case were multifaceted. Initially misdiagnosed with MS due to the clinical presentation of optic neuritis and subsequent central nervous system (CNS) involvement, the patient’s condition did not respond to traditional MS treatments such as azathioprine. Rituximab is a well-established preventive therapy for NMOSD [1]. However, the persistence of neurologic symptoms coupled with the emergence of petechial rashes on both lower extremities raised the suspicion of an underlying hematologic disorder, necessitating further investigation.

A comprehensive search for randomized controlled trials reporting the use of low-dose (100 mg) or standard-dose (375 mg/m^2^) rituximab in ITP treatment was conducted [2]. The administration of rituximab, a chimeric monoclonal antibody targeting CD20-positive B lymphocytes, marked a turning point in this patient’s treatment. The treatment of acute and recurrent attacks in NMOSD is based upon the evidence that humoral autoimmunity plays a role in the pathogenesis of NMOSD [4]. It was initiated at a dose of 1 g on day 1 and day 15, as commonly employed in induction treatment for NMOSD. NMOSD is a rare autoimmune inflammatory demyelinating disease of the CNS [4].

Further complicating the diagnosis was the patient’s history of other medical conditions, including gastric ulcers from *H. pylori* infection and rectosigmoid adenocarcinoma. *H. pylori* infection has been implicated in the pathogenesis, including cardiovascular, hematologic, and autoimmune diseases [5]. One hypothesis regarding the mechanism by which *H. pylori* induces the development of ITP is the cross-reactive antibodies that are produced that react with both *H. pylori* components and platelet surface antigens through molecular mimicry [5]. Other malignancies that were recorded to be related to NMOSD are the breast and ovary in women, while in men, lung, and prostrate predominate [6]. These comorbidities added a layer of complexity to the clinical picture and underscored the importance of a comprehensive assessment to unravel the interplay between neurologic and hematologic disorders.

The hematologic aspect of this case became apparent when thrombocytopenia was identified, with platelet counts as low as 4 × 10^9^/L.

ITP emerged as a plausible diagnosis, although the etiology remained elusive, as is often the case with ITP. The patient’s rapid and remarkable response to pulse therapy with intravenous methylprednisolone, leading to a significant increase in platelet counts and resolution of petechial rashes, provided crucial diagnostic and therapeutic insights.

The first study of rituximab showed that most peripheral blood B cells were depleted until 14 months [7]. Notably, the patient exhibited improved motor strength in all extremities following rituximab therapy, leading to a reduction in her EDSS score from 7.0 to 6.0.

**Figure 4:**
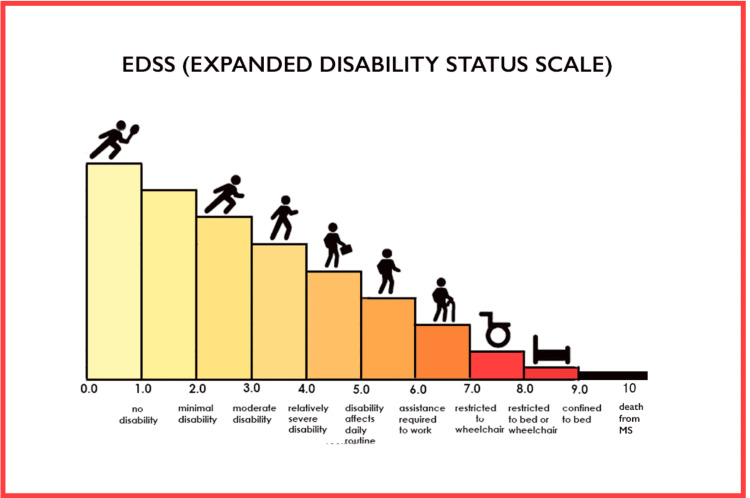
The Expanded Disability Status Scale (EDSS) focuses mainly on the patient’s ability to walk.

## Conclusions

This unique case underscores the challenges and complexities associated with the diagnosis and management of combined neurologic and hematologic disorders. The coexistence of NMOSD and ITP in this patient, along with the absence of AQP4-IgG seropositivity, highlights the clinical heterogeneity of NMOSD and the necessity of a broad diagnostic approach.

Prompt recognition of the hematologic component, namely, ITP, was pivotal in this case. The subsequent initiation of rituximab therapy not only resulted in hematologic improvement but also contributed to enhanced motor function.

The case sheds light on the evolving treatment landscape for NMOSD and highlights the potential utility of rituximab in managing this complex disorder. Further research and case studies are warranted to better understand the underlying mechanisms and optimal treatment strategies for patients with similar presentations, ultimately improving their quality of life and prognosis.
